# Slow-release nitrogen fertilizer application regulated rhizosphere microbial diversity to increase maize yield

**DOI:** 10.3389/fpls.2024.1481465

**Published:** 2024-11-22

**Authors:** Tiantian Meng, Jingjing Shi, Xiangqian Zhang, Xiaoqing Zhao, Dejian Zhang, Liyu Chen, Zhanyuan Lu, Yuchen Cheng, Yonghe Hao, Xiaoyu Zhao, Yu Wang

**Affiliations:** ^1^ College of Agriculture, Hebei Agricultural University, Baoding, China; ^2^ College of Life Sciences, Inner Mongolia University, Hohhot, China; ^3^ Institute of Plant Protection, Inner Mongolia Academy of Agricultural and Animal Husbandry Sciences, Hohhot, China; ^4^ Key Laboratory of Ecological Restoration and Pollution Prevention of Degraded Farmland of Inner Mongolia Autonomous Region, Inner Mongolia Academy of Agricultural and Animal Husbandry Sciences, Hohhot, China; ^5^ Erdos Agricultural and Animal Husbandry Technology Extension Center, Ordos Agriculture and Animal Husbandry Bureau, Ordos, China

**Keywords:** nitrogen fertilizer application rate, maize, microbial diversity, co-occurrence network, yield

## Abstract

The one-time application of slow-release nitrogen fertilizer can not only reduce the labor input, but also reduce the mechanical input cost, and has the characteristics of slow release and reduce volatilization loss. This research is grounded in a localization trial initiated in 2018, which underwent comprehensive analysis utilizing high-throughput sequencing technology to elucidate the mutual feeding mechanism of slow-release nitrogen fertilizer application rate on microbial community structure, network complexity, and maize yield in different root niches (bulk soil, rhizosphere, and endosphere). Soil characteristics, microbial community composition, and collinear network of different ecological niches under slow-release nitrogen fertilizer were analyzed, and the key core species affecting the stability of the microbial network and the factors driving yield were identified. The results showed that nitrogen application increased the diversity of bacteria, and nitrogen application significantly increased the diversity of rhizosphere bacteria and fungi due to rhizosphere effects. Slow-release nitrogen fertilizer increased the complexity of the bacterial network and decreased the complexity of the fungal network, particularly, the network complexity of bacteria and fungi in the rhizosphere was higher than that in the bulk soil and the rhizosphere. The application of slow-release nitrogen fertilizer increased the abundance of Proteobacteria, Bacteroidota, Gemmatimonadota, Actinobacteria, Ascomycota, Basidiomycota and other dominant bacteria. Coordinate soil physical and chemical properties, increase soil enzyme activity and soil nutrients, improve soil microenvironment, regulate microbial community composition, and promote above-ground yield increase, in which nitrogen application, urease, nitrate reductase and nitrate nitrogen are the main driving factors for yield increase. These findings provide a new idea for the mutual feeding mechanism of slow-release nitrogen fertilizer on microbial diversity and yield in different ecological niches. To selection of suitable nitrogen application rate and regional ecological security in the agro-pastoral ecotone.It offers a theoretical framework for establishing optimal nitrogen application rates and ensuring food security in agro-pastoral ecotones.

## 1 Introduction

Maize (*Zea mays* L.), one of the world’s three primary grain crops, is widely distributed and highly productive. It is a crucial crop for grain, economic value, and animal feed, significantly contributing to China’s economic growth and food security (Zhang et al., 2021). The United States and China are the world’s largest maize consumers, together accounting for 51.6% of global maize consumption. In 2023, China imported 27.13 million tons of maize, marking a 31.6% increase from the same period in 2022, highlighting the critical need to enhance maize production (Zhao and He, 2024). Nitrogen is the primary element influencing maize yield, and an adequate nitrogen supply ensures higher economic crop yields (You et al., 2023). Compared to conventional urea, the nutrient release profile of slow-release fertilizers aligns with the nutrient demands of crops. A single basal application of slow-release nitrogen can meet the nutrient requirements of crops throughout the growth period, support high-quality crop growth (Chen J. et al., 2023), and reduce nitrogen volatilization and nitrate leaching losses. Slow-release nitrogen enhances the absorption and utilization efficiency of nitrogen by crops, allowing for a reduction in the amount of nitrogen applied (Xing et al., 2015). Excessive nitrogen application surpasses the economic optimal rate, rapidly diminishing fertilizer efficiency and significantly increasing residual nitrogen in the soil and environmental losses (Feng et al., 2022). The development and implementation of scientific fertilization technologies are crucial for ensuring stable maize yields, maintaining soil nutrients, and promoting sustainable agricultural development.

Nitrogen application can regulate soil enzyme activity by influencing soil chemical properties and microbial diversity, thereby impacting crop yield formation (Yang et al., 2022). As the nitrogen application rate and inorganic nitrogen content increase, slow-release nitrogen fertilizers can maintain soil inorganic nitrogen levels throughout the maize growth period, reduce ammonium nitrogen nitrification (Huang et al., 2018), and regulate soil nutrient changes (Wang et al., 2024). Nitrogen fertilizer application rate enhanced the activities of soil urease, protease (Liu Y. et al., 2023), and nitrate reductase, leading to significant changes in soil properties (such as organic carbon, inorganic nitrogen, and pH) and microbial biomass carbon and nitrogen (Tang et al., 2021). Comprehensive analysis indicates that appropriate nitrogen fertilizer application rates can promote soil nitrite reductase and nitrate reductase activities, increase nitrogen conversion efficiency, and subsequently enhance yield (Wang et al., 2023), The optimum nitrogen application rate in agro-pastoral areas is 200-240 kg·ha^-1^ (Chang et al., 2013; Fu X. et al., 2017).

Microorganisms function as decomposers. In nutrient biogeochemical cycles, soil microorganisms are crucial for nitrogen conversion and absorption, degrading harmful soil substances and providing essential elements for plant growth (Xiong et al., 2014). The soil microbiome is a vital source of the rhizosphere microbiome, with endosphere microorganisms potentially colonizing under specific conditions. The root spatial ecological niche is a key driver of the root microbiome (Ren et al., 2020) The rhizosphere refers to the narrow region of soil influenced by plant roots within a few millimeters. This region differs from bulk soil in physical, chemical, and biological properties and is characterized by close soil-root-microbial interactions (Venturi and Keel, 2016). Soil chemical properties, pH, and other factors influence the rhizosphere microbial community structure by affecting plant physiological traits and rhizosphere sediment composition (Philippot et al., 2013). It is influenced by soil and environmental conditions, forming specific micro-ecosystems (Xu et al., 2014). Nitrogen application can alter the composition and function of microbial communities by modifying soil physical and chemical properties and the microenvironment, thereby impacting plant growth (Li et al., 2020), This effect may be due to nitrogen application lowering soil pH, which in turn reduces microbial diversity and function (Zhou et al., 2020). That short-term nitrogen fertilizer application rate had a greater impact on fungal communities compared to bacterial communities within different spatial structures of silage maize, with increased nitrogen application weakening fungal community interactions (Bai et al., 2022b). Therefore, nitrogen application rates can influence soil microbial diversity and community composition. Appropriate nitrogen application rate not only increases the number of fungi and bacteria in the soil but also regulates soil nitrogen transformation (Fu Z. et al., 2017). However, excessive application of nitrogen fertilizer not only reduces the diversity of rhizosphere fungi but also alters the structural composition of the community (Zhou et al., 2016). Currently, most studies focus on the effects of fertilization on rhizosphere soil microbial diversity, with few investigating the changes in ecological niche diversity within different root spaces of maize fields.

Due to the extensive use of nitrogen fertilizers and severe pollution in the agro-pastoral ecotone of Inner Mongolia, this study, based on a 2018 experiment, investigated soil characteristics, microbial diversity across different ecological niches, collinearity networks, and the regulatory mechanisms of community composition and yield under varying slow-release nitrogen fertilizer application rates. The objectives were to elucidate (1) the effects of varying nitrogen application rates on bacterial diversity in different root niches (bulk soil, rhizosphere, endosphere); (2) the impacts of nitrogen application on the complexity and stability of bacterial and fungal networks in various ecological niches; (3) to determine the optimal nitrogen application rate for maize fields in the agro-pastoral ecotone.

## 2 Materials and methods

### 2.1 Study site description

**Figure 1:**
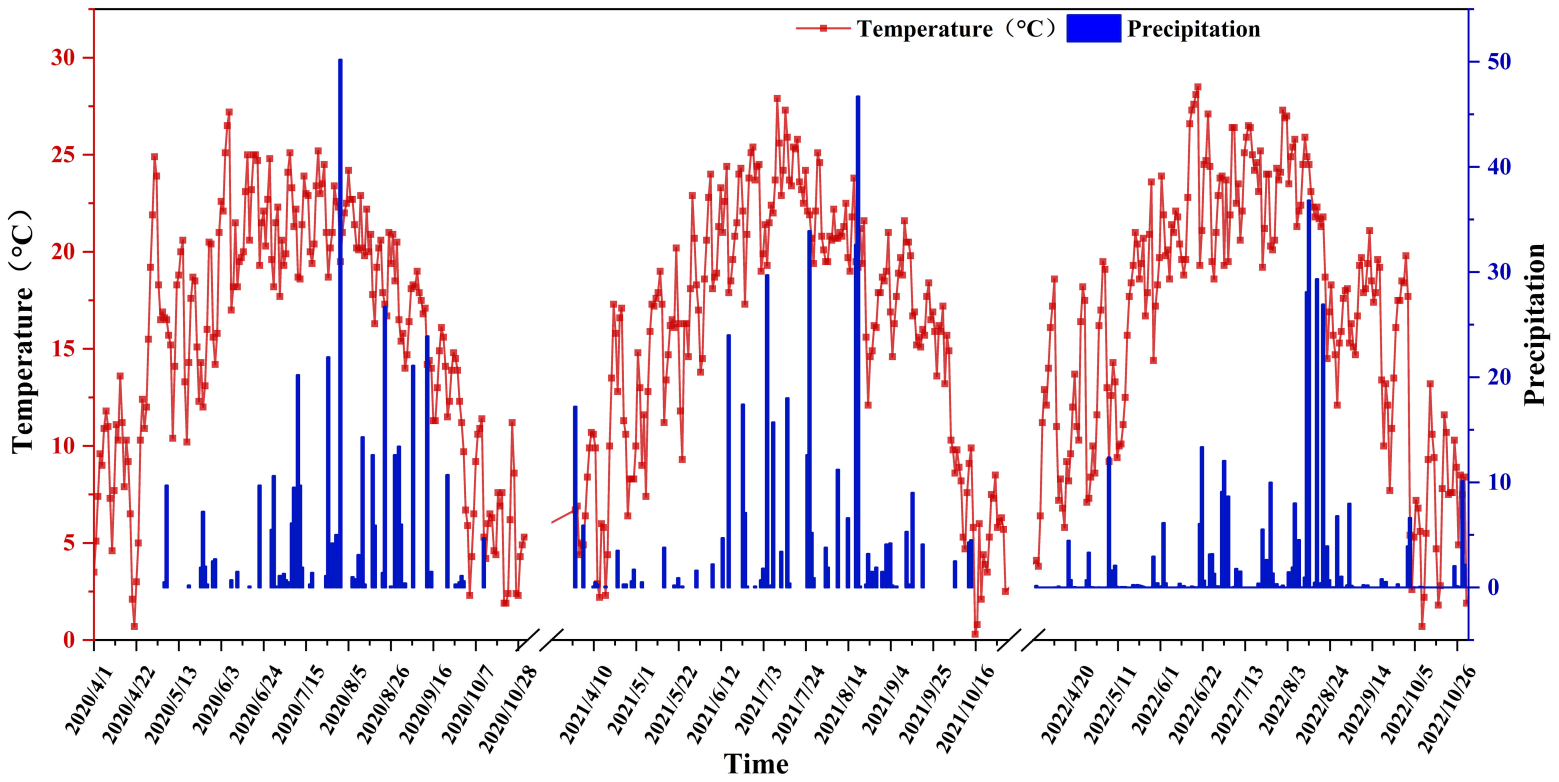
Average temperature and precipitation in Hohhot in 2020-2022.

This research is carried out at the experimental base of the Academy of Agricultural and Animal Husbandry Sciences in the Inner Mongolia Autonomous Region (40°46’ N, 111°30’ E, altitude 1040 m). Situated in the northern part of North China and the southern region of the Daqing Mountains, this area exemplifies the typical continental climate of the Mongolian Plateau. Spring in this region is characterized by dryness and strong winds, accompanied by significant fluctuations in temperature. Summers are brief, marked by high temperatures and sparse rainfall, with precipitation and heat occurring simultaneously. Autumn experiences a rapid decline in temperature, frequently leading to frost. Winters are prolonged, cold, and receive minimal snowfall. For a detailed overview of the entire growth period of maize in 2022, refer to **Figure 1**. The soil type at the study site was loam, and the preceding crop was maize. The soil at the site contained 17.68 g·kg^-1^ of organic matter, 1.12 g·kg^-1^ of total nitrogen, and 0.57 g·kg^-1^ of total phosphorus. Additionally, it had 49.25 mg·kg^-1^ of alkali-hydrolyzed nitrogen, 28.70 mg·kg^-1^ of available phosphorus, and 93.58 mg·kg^-1^ of available potassium. The maximum field moisture content ranged from 23% to 25%, with a pH value of 7.89, and the bulk density of the 0 – 20 cm soil layer was 1.18 g·cm^-3^.

### 2.2 Experimental design

This experiment is built upon a positioning study initiated in 2018. A randomized block design is implemented, and “Guangde No. 5” is chosen as the test variety. Six nitrogen fertilizer application rates are establishing: 0 kg·ha^-1^ (N0), 120 kg·ha^-1^ (N8), 180 kg·ha^-1^ (N12), 240 kg·ha^-1^ (N16), 300 kg·ha^-1^ (N20), and 360 kg·ha^-1^ (N24). Each treatment is replicated three times, resulting in a total of 18 plots, each covering an area of 27.9 m². Sowing is performed with equal row spacing, maintaining a row spacing of 0.6 m and a plant spacing of 22.2 cm. Two protective lines of 1 m each are set between the plots. Sowing is done manually, with a seeding density of 75,000 plants per hectare. Each treatment received the same dose of phosphate fertilizer (300 kg·ha^-1^ (NH_4_)_2_HPO_4_) and potassium fertilizer (120 kg·ha^-1^ K_2_SO_4_). Slow-release nitrogen fertilizer is applied using resin-coated urea containing 46% nitrogen, with a particle diameter ranging from 2 mm to 4.75 mm (China Coal Energy Co., LTD.). All fertilizers are applied once as a base fertilizer before sowing, with no additional fertilization during later stages. Manual weeding and pest control are performed during the maize growing stage. The irrigation method employed is drip irrigation, with the main pipe having a diameter of 60 mm. The irrigation volume is monitored using a water meter. The drip irrigation volumes for June, July, and August were 425.00 m³·ha^-1^. Other field management practices are consistent with standard field management procedures.

### 2.3 Soil chemical character, biological character determination

Soil samples are collected during the tasseling stage of maize. The microbial biomass carbon and nitrogen in soil are quantified using the chloroform fumigation-leaching method (Wilson, 1988; Brookes et al., 1985) Soil inorganic nitrogen (NO_3_^–^N, NH_4_^+^-N) is measured via the colorimetric method, while the leachate is analyzed using a flow analyzer (Zhao et al., 2014). Soil organic carbon and organic matter contents were determined through the potassium dichromate oxidation method (Bao, 2008). The soil pH value is measured using a pH meter (pH-3C, REX, Shanghai) following a 1:5 soil-to-water mixture. The activities of soil urease, nitrite reductase, nitrate reductase, protease, and hydroxylamine reductase are all assessed using the microporous method with reagents from the detection kit of Suzhou Yanxi Biotechnology Co., LTD., and the TECAN/Decon GENios Plus multifunctional fluorescent enzyme marker.

### 2.4 Maize yield determination

At the mature stage of maize, two rows were randomly selected for each nitrogen application level. Ten maize plants from the middle of each row are chosen, totaling 20 plants per treatment, which are replicating three times. The plants are then air-dried and threshed to measure yield.

### 2.5 Microbial diversity analysis

#### 2.5.1 Sample collection

Soil samples and root systems are collected during the maize decamping and silking stages. In each plot, soil cores (0 – 20 cm) are taken from five sample points along an “S” shaped curve and combined to form a representative sample of the plot. The soil samples are divided into two parts. One part is dry and screened for the determination of physical and chemical properties, while the other part is wet soil, screened through 2mm sieve, and stored at -20°C for the determination of microbial biomass carbon and nitrogen, nitrate, and ammonium nitrogen. A typical maize plant is selected, the entire root system is excavated, and the bulk soil surrounding the root is shaken off and sieved through 1 mm screen before being placed in a sterile centrifuge tube. The soil within 2mm of the roots is shaken off, sieved through 1 mm screen as rhizosphere soil, and placed in a sterile centrifuge tube. Different parts of the maize roots are evenly cut, washed with pure water, stored in sterile centrifuge tubes, frozen with liquid nitrogen, and kept at -80°C for 16S and ITS amplification sequencing to determine microbial diversity.

#### 2.5.2 Genomic DNA extraction

The genomic DNA of 10 g samples is extracted by DNA extraction kit, and the DNA concentration is determined by agarose gel electrophoresis and NanoDrop2000.

#### 2.5.3 PCR amplification and database construction

Genomic DNA is used as a template, and specific primers with barcodes are employed for PCR amplification of the selected sequencing region to ensure high efficiency and accuracy. The bacterial 16S gene amplification primer set consisted of 343F (5’-TACGGRAGGCAGCAG-3’) and 798R (5’-AGGGTATCTAATCCT-3’), while the fungal ITS base amplification primer set included ITS1F (5’-CTTGGTCATTTAGAGGAAGTAA-3’) and ITS2 (5’-GCTGCGTTCTTCATCGATGC-3’) (Wu et al., 2015; Xiong et al., 2012). After electrophoresis detection and magnetic bead purification, a two-round PCR is performed using the purified products as templates. Following another round of purification, Qubit quantification is conducted on the resulting PCR products before pooling equal amounts for sequencing. The original sequencing data are in FASTQ format and are processed by Cut adapt software to remove primers from the raw sequences. DADA2 is then used to perform quality control analyses such as filtering, denoising, merging paired-end reads, and removing chimeras based on default parameters in the QIIME 2 software package to obtain representative sequences and an ASV abundance table. Representative sequences are annotated against databases using the Silva database version 138 for 16S comparison or the Unite database for ITS comparison via the q2-feature-classifier software under default parameter settings (Bolyen et al., 2019; Callahan et al., 2016).

### 2.6 Analytical methods

Data collection and collation were performed using Excel 2021 (Microsoft Corp., Redmond, WA, USA). SPSS 25 (SPSS Inc., Armonk, NY, USA) is employed to analyze the differences in soil physicochemical properties, microbial biomass carbon and nitrogen, soil enzyme activity, and yield under each nitrogen application rate using ANOVA (*P* < 0.05). The α-diversity indices (Chao index, Shannon index) of soil samples are calculated using QIIME software. Principal coordinate analysis (PCoA) and PERMANOVA (Bray-Curtis distance) are utilized to analyze the bacterial β-diversity under different treatments. Sequencing data are analyzed online using the Majorbio i-Sanger cloud platform (www.i-sanger.com).

The Spearman correlation matrix is calculated using the “hmisc”, “psych”, and “igraph” packages in R software to construct the co-occurrence network. The P values of the correlation matrix are adjusted using the Benjamini-Hochberg method. The Spearman correlation is calculated using significant pairwise ASV correlations (*P* < 0.05, R > 0.5) to construct the network. The relative absolute value threshold is set to 0.6 with *P* < 0.05. The resulting correlations are imported into the Gephi platform and visualized using the Fruchterman-Reingold algorithm (Bastian et al., 2009). The average clustering coefficient, average path length, and modularity of the network are then calculated (Newman, 2006). Based on the network analysis, the top 20 network hubs under nitrogen treatment are selected, and the key species are classified. The Zi and Pi values of network nodes are calculated using R’s “igraph” package, and nodes are categorized into four types based on their topological characteristics: (1) Module hubs, centers of modules, nodes with high connectivity within modules, Zi > 2.5 and Pi < 0.62; (2) Connectors, inter-module connection nodes, nodes with high connectivity between modules, Zi < 2.5 and Pi > 0.62; (3) Network hubs, nodes with high connectivity in the entire network, Zi > 2.5 and Pi > 0.62; (4) Peripherals, nodes with low connectivity within or between modules, Zi < 2.5 and Pi < 0.62 (Olesen et al., 2007). Module hubs, connectors, and network hubs are generally regarded as key nodes. Core species play a crucial role in maintaining the stability of the network structure (Deng et al., 2012). The key driving factors of yield are analyzed using the “Random Forest” package in R software, and the Mantel test is conducted using the “vegan” package in R software.

## 3 Results

### 3.1 Effect of slow-release nitrogen fertilizer application rate on maize yield

**Figure 2:**
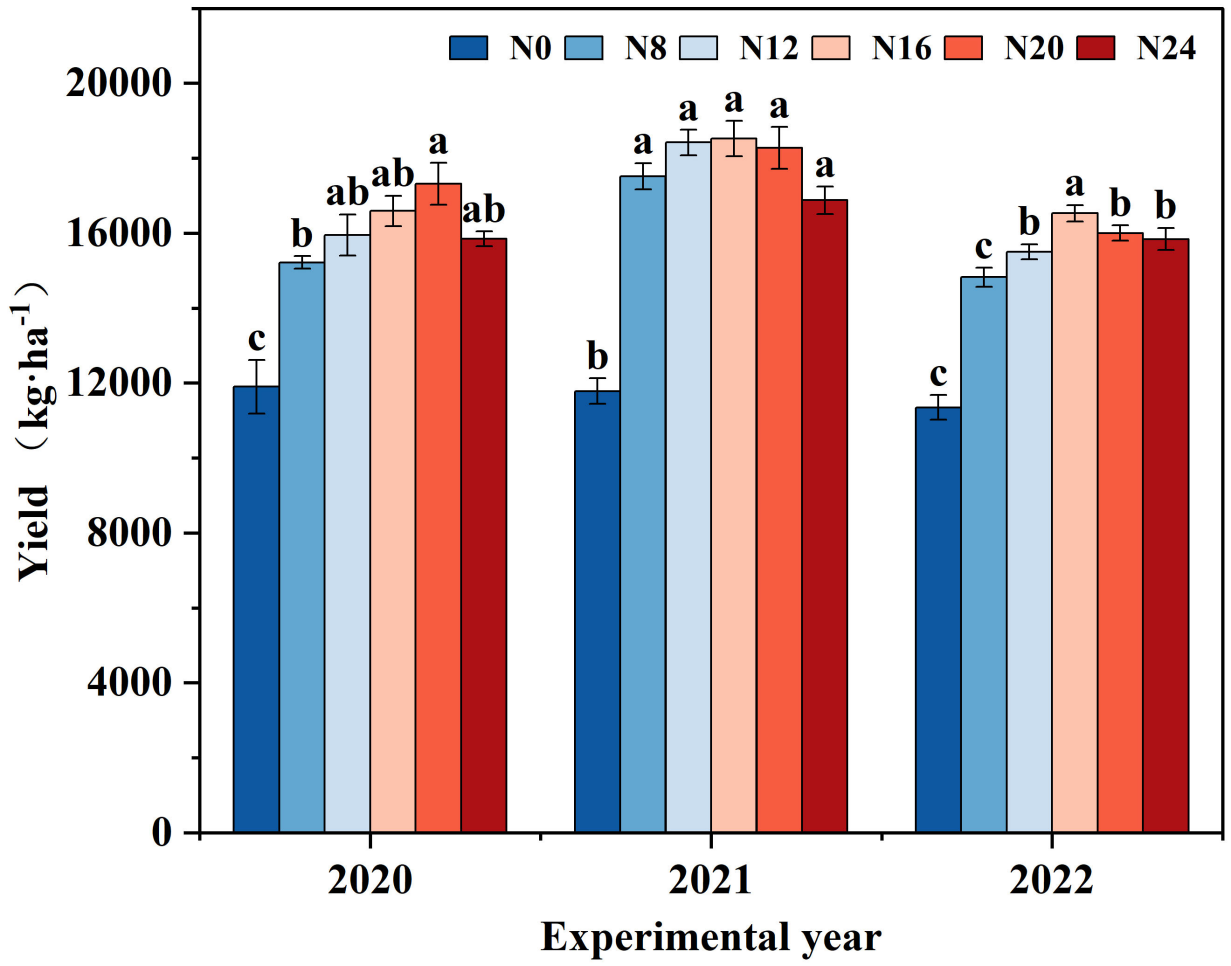
Yield of maize under different nitrogen fertilizer application rates. 0 kg·ha^-1^(N0); 120 kg·ha^-1^(N8); 180 kg·ha^-1^(N12); 240 kg·ha^-1^(N16); 300 kg·ha^-1^(N20); 360 kg·ha^-1^(N24). The distinct lowercase letters indicate statistically significant differences at the 0.05 significance level.

The variations in maize yield under diverse nitrogen application rates from 2020 to 2022 were analyzed (**Figure 2**), and the tendency of yield alteration was consistent across different years. With the escalation of the nitrogen application rate, the yield initially rose and subsequently declined. The yield in 2020 peaked in N20 (300 kg·ha^-1^), while the yield in 2021 and 2022 reached the maximum in N16 (240 kg·ha^-1^), presenting significant differences compared with N0. The yield of each nitrogen application treatment in 2021 surpassed that in 2020 and 2022, which might be associated with the abundant rainfall in 2021. Maize yield ranged from 11348.02 to 18526.47 kg·ha^-1^ from 2020 to 2022, and the disparity between nitrogen application treatments and N0 achieved a significant level (*P* < 0.05).In 2020, N8, N12, N16, N20, and N24 treatment yields increased by 27.89%, 34.02%, 39.42%, 45.49%, and 33.15%, respectively, compared to N0 (CK); and in 2020, N8, N12, N16, N20, and N24 treatments yields increased by 48.63%, 56.30%, respectively, compared to N0 (CK), 57.19, 55.06, and 43.19 percent; and the yields of N8, N12, N16, N20, and N24 treatments increased by 30.63, 36.61, 45.69, 40.99, and 39.61 percent, respectively, compared to N0 (CK) in 2020, indicating that nitrogen application significantly affects yield changes, indicating that nitrogen application would significantly influence yield variation.

### 3.2 Effects of slow-release nitrogen fertilizer application rate on soil chemical and microbial properties

In this study, fertilization was found to significantly increase soil NO_3_^–^N and NH_4_^+^-N contents. SOC, MBC, and MBN contents initially increased and then decreased with higher nitrogen application rates, reaching their peak under the N16 treatment. These values were significantly higher than those in other treatments (*P* < 0.05). The application of nitrogen fertilizer reduced soil pH, with significant decreases observed as nitrogen application rates increased (**Table 1**). The N0 treatment exhibited the highest pH value (8.25). Additionally, nitrogen fertilizer application rate increased soil nutrient content and fertility compared to no fertilization. Changes in soil enzyme activity under different nitrogen application levels were further investigated. The application of slow-release nitrogen fertilizer influenced soil enzyme activity. Nitrate reductase and nitrite reductase, alkaline protease, hydroxylamine reductase, and urease activities were significantly increased and tended to increase and then decrease with increasing nitrogen application compared to N0 (**Table 1**), reaching their highest values under the N16 treatment (240 kg·ha^-1^). Beyond this level, enzyme activity began to decline with further increases in nitrogen application. These results indicate that an optimal nitrogen supply can enhance soil nutrient availability, provide sufficient substrates for microorganisms, and promote nitrogen cycling.

**Table 1:** PERMANOVA analysis of bulk soil, rhizosphere and endosphere microorganisms with different nitrogen application rates.

Factors	Bacterial community		Fungal Community	
	*R^2^*	*p*-value	*R^2^*	*p*-value
**Bulk soil**	0.407	0.001	0.532	0.001
**Rhizosphere**	0.397	0.001	0.55	0.001
**Endosphere**	0.409	0.001	0.502	0.001
**Nitrogen fertilizer application rate**	0.114	0.112	0.172	0.003
**Ecological niche**	0.322	0.001	0.352	0.001

### 3.3 Response of microbial community diversity to nitrogen application rate

**Figure 3:**
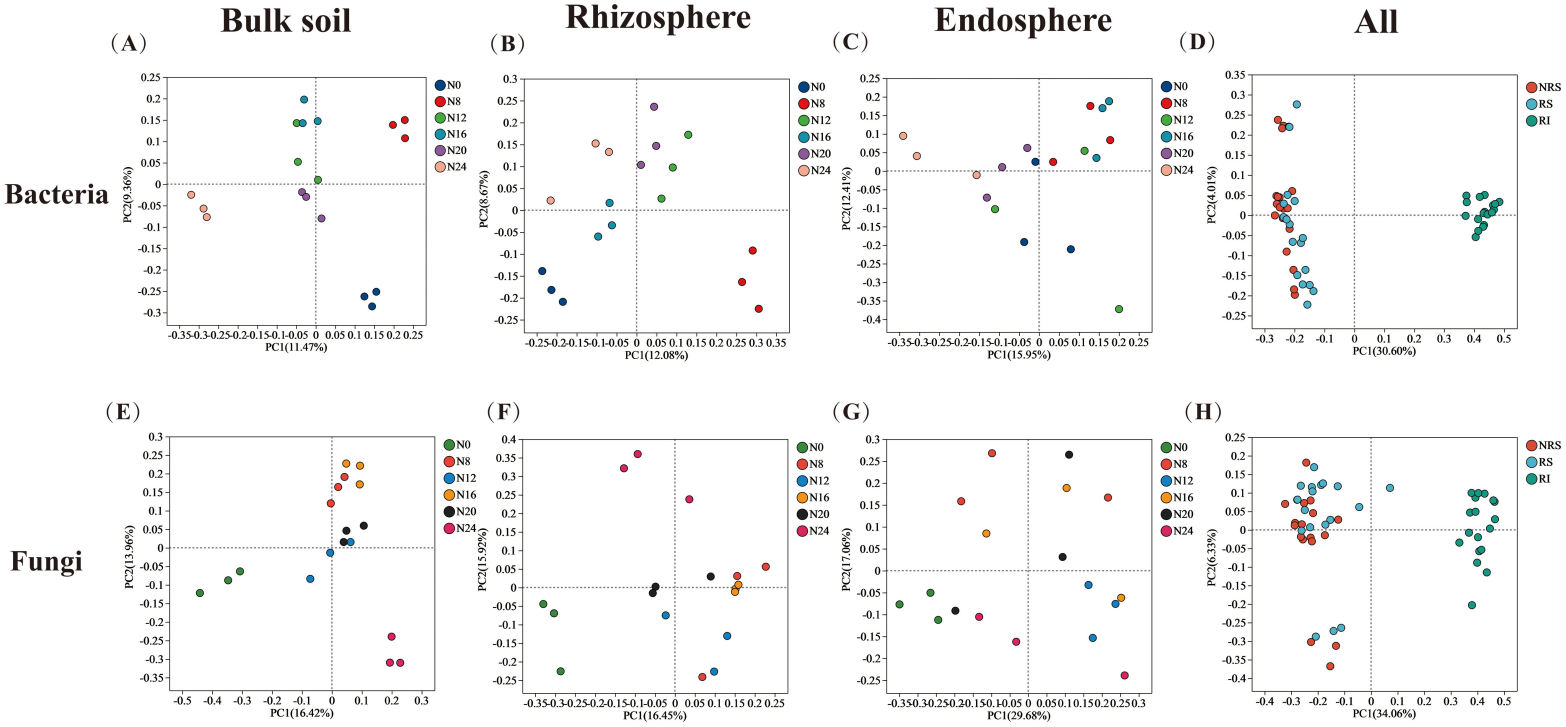
PCoA analysis of different nitrogen fertilizer application rate. β-diversity differences in bulk soil, rhizosphere, and endosphere of bacteria **(A–D)** and fungi **(E–H)** in different ecological niches and at all locations. NRS, bulk soil; RS, rhizosphere; RI, endosphere.

In this study, we investigated the changes in α-diversity of microorganisms across different spatial niches under varying nitrogen application levels. We found that the species richness and diversity of bacterial and fungal communities in endosphere were lower than those in rhizosphere and bulk soils. The application of nitrogen fertilizer significantly increased the Chao and Shannon indices of bulk soil, rhizosphere soil, and endosphere bacterial communities. It also enhanced the Chao and Shannon indices of rhizosphere fungi, while decreasing the Chao index of bulk soil fungal communities (**Supplementary Table S1**). The results demonstrated that fertilization increased the diversity of bacterial communities and decreased the diversity of bulk soil fungi. Nitrogen application particularly affected the rhizosphere bacterial and fungal microbial populations significantly, bacterial populations under bacterial N8, N12, N16, N20, N24 treatments increased by 103.85%, 101.51%, 17.48%, 74.56%, 23.83%, and fungal N8, N12, N16, N20, N24 treatments increased by 23.52%, 0.79%, 7.35%, 0.51%, -2.76%, respectively, compared to the N0 treatment. Increased by 23.52, 0.79, 7.35, 0.51, -2.76%, respectively (**Supplementary Table S1**). Principal coordinate analysis (PCoA) was used to compare the overall differences in soil bacterial and fungal communities across different treatments and ecological niches (**Figure 3**). ANOSIM (ASV level) indicated significant differences in the community structure of bacteria and fungi across different spatial locations (*P* < 0.001), and notable differences in bulk soil, rhizosphere soil, and endosphere (*P* < 0.01). Different nitrogen application rates significantly altered the community structure of bacteria and fungi (*P* < 0.05), with the most pronounced effects observed in rhizosphere soil. PERMANOVA analysis (ASV level) confirmed that niche variation (bulk soil, rhizosphere, and root endosphere) was the primary factor influencing bacterial and fungal community diversity (*P* < 0.001), with a greater effect on fungi than on bacteria. Nitrogen treatment had no significant effect on bacterial communities but significantly affected fungal communities. Nevertheless, PERMANOVA analysis of different ecological niches revealed that varying nitrogen application rates significantly impacted community diversity within bacterial and fungal endospheres (*P* < 0.001) (**Table 2**).

**Table 2:** Effects of different nitrogen fertilizer application rate on soil chemical and biological characteristics.

Soil properties	N0	N8	N12	N16	N20	N24	F-value
pH	8.25 ± 0.04a	8.15 ± 0.04b	8.15 ± 0.02b	8.15 ± 0.0b	8.11 ± 0.02b	8.05 ± 0.02c	12.966^***^
SOC (g·kg^-1^)	11.52 ± 0.69c	12.63 ± 1.01b	13.07 ± 0.06b	15.29 ± 0.18a	13.41 ± 0.57b	13.3 ± 0.76b	11.139^***^
NH_4_^+^-N (mg·kg^-1^)	5.62 ± 0.21e	5.82 ± 0.06de	5.99 ± 0.09cd	6.05 ± 0.08c	6.36 ± 0.11b	6.91 ± 0.16a	34.594*^**^
NO_3_^–^N (mg·kg^-1^)	7.46 ± 0.07e	9.13 ± 0.31d	8.99 ± 0.05d	9.67 ± 0.07c	11.32 ± 0.02b	26.17 ± 0.24a	5044.785^***^
MBC (mg·kg^-1^)	323.6 ± 19.41c	346.69 ± 5.41b	388.86 ± 1.52a	400.99 ± 7.74a	365.01 ± 5.28b	359.03 ± 2.75b	28.167^***^
MBN (mg·kg^-1^)	69.51 ± 1.52d	73.72 ± 1.35c	75.32 ± 2.15bc	80.06 ± 1.45a	77.45 ± 1.26b	74.86 ± 0.54c	17.774^***^
S-NiR (umol·(d^-1^·g^-1^))	0.99 ± 0.13d	1.54 ± 0.31c	2.09 ± 0.02c	2.21 ± 0.08a	1.66 ± 0.11b	1.622 ± 0.2c	18.826^***^
S-NR (ug·(d^-1^·g^-1^))	10.3 ± 1.27d	12.6 ± 0.64c	13.37 ± 0.37b	15.52 ± 0.95a	14.58 ± 0.57bc	12.621 ± 0.16bc	17.066^***^
ALPT (mg·(d^-1^·g^-1^))	0.1155 ± 0.011c	0.3489 ± 0.053b	0.4149 ± 0.011a	0.5847 ± 0.053a	0.297 ± 0.010b	0.1792 ± 0.03b	74.234^***^
HR (ug·(d^-1^·g^-1^))	997.34 ± 7.21d	1023.86 ± 12.94c	1050.81 ± 3.54bc	1092.78 ± 25.66a	1043.95 ± 18.43b	1043.145 ± 25.27c	9.601^**^
UE (ug·(d^-1^·g^-1^))	1768.88 ± 32.1e	2115.97 ± 92.13c	2344.54 ± 98.54b	2539.25 ± 286.3a	2255.65 ± 48.31c	2285.289 ± 58.15d	11.357^***^

SOC, soil organic carbon; MBC, microbial biomass carbon; MBN, microbial biomass nitrogen; NO_3_–N, nitrate nitrogen; NH_4_^+^-N, ammonium nitrogen; S-NiR, Soil enzymes include soil nitrite reductase; S-NR, soil nitrate reductase; alkaline protease; HR, hydroxylamine reductase; UE, urease. ** means P<0.01; *** means P<0.001.

The dominant bacteria in bulk soil and rhizosphere soil under different nitrogen application treatments were Proteobacteria, Gemmatimonadota, and Actinobacteria (**Supplementary Figures S2A, B**). The dominant fungal phyla were Basidiomycota, Ascomycota, and unclassified_k:Fungi (**Supplementary Figures S2D, E**). The dominant bacteria in the endosphere under different nitrogen application treatments were Proteobacteria, Actinobacteria, and Bacteroidota (**Supplementary Figure S2C**). The dominant fungal phyla were Ascomycota, Olpidiomycota, and Glomeromycota (**Supplementary Figure S2F**). The abundance of Gemmatimonadota in endosphere bacteria under N8-N24 treatments was higher than in the absence of nitrogen (N0), while the difference was not significant in bulk soil. The abundance of Basidiomycota was higher in bulk soil and endosphere bacteria under N8-N24 treatments compared to no nitrogen application (**Supplementary Figure S2**).

### 3.4 Response of microbial network analysis to nitrogen application rate

**Figure 4:**
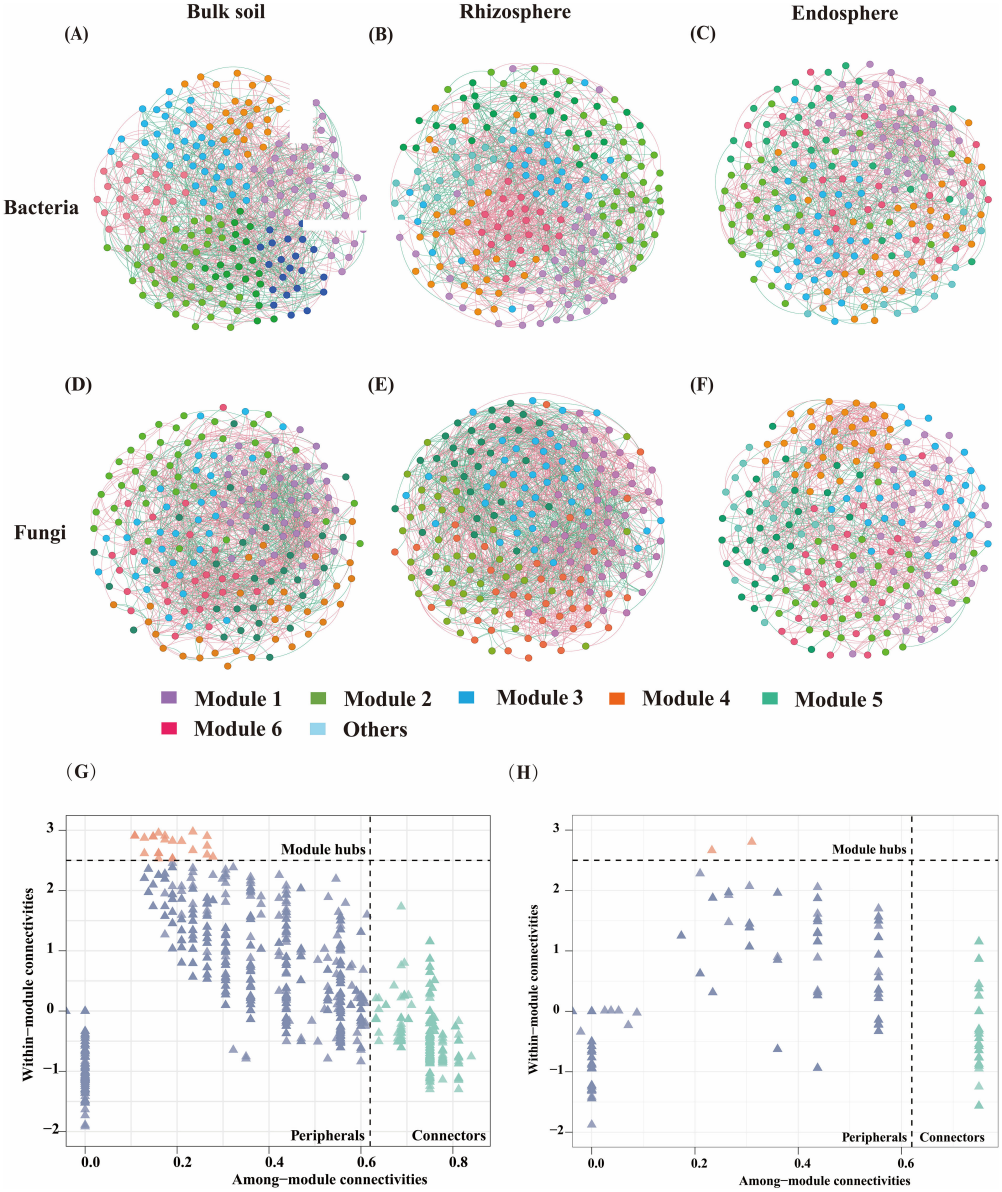
ASVs co-occurrence network of bacterial and fungal communities based on correlation analysis. For each network, the nodes are colored by module, and the width of the edge is proportional to the Spearman correlation coefficient. The color of the edge indicates the relationship between the nodes. The red side shows a positive correlation and the green side shows a negative correlation. **(A-D)** represents bacterial networks in Bulk soil, Rhizosphere and Endosphere. **(E-H)** represents the fungal network of bulk soil, rhizosphere, and endosphere.

ASV correlation networks were utilized to analyze combination patterns across different nitrogen application rates and spatial locations. It was revealed that the complexity of the rhizosphere network surpassed that of the endosphere and bulk soil in ASV co-occurrence networks across bulk soil, rhizosphere soil, and endosphere bacterial and fungal communities (**Figure 4**). In the bacterial network, bulk soil exhibited the highest number of edges (1131) and negatively correlated edges (44.92%). The modularity index and clustering coefficient of the endosphere bacterial network were higher than those of the rhizosphere and bulk soil soils. Among the fungal networks, the rhizosphere microbial network was more complex and exhibited the largest number of edges (1815), particularly negatively correlated edges (39.18%). The modularity index, clustering coefficient, and average degree of rhizosphere fungi were higher than those of bulk soil and roots (**Supplementary Table S2**).

Since microbial and rhizosphere turnover in endosphere and bulk soil are interrelated processes, samples from the three sites were integrated into a whole community for network analysis across different treatment groups. Nitrogen application increased network complexity, especially in the N12 (node 198, edge 5699) treatment, where bacterial network complexity was significantly higher than in the N0 (node 196, edge 4485) treatment, and fungal network complexity (node 200, edge 3079) was higher than in the no N treatment (node 199, edge 2941) (**Supplementary Figure S1**, **Supplementary Table S3**). The average path length, clustering coefficient, and modularity value of bacterial networks under different nitrogen application treatments were lower than those of fungi, although the modularity index and clustering coefficient were higher (**Supplementary Table S3**). In the bacterial network, fertilization treatment exhibited higher complexity and connectivity than the control treatment, along with a higher average degree (57.566) and more negatively correlated edges (48.03%) (**Supplementary Table S3**, **Supplementary Figure S1**). Bacterial network, showing an initial increase followed by a decrease with higher nitrogen application rates. In the fungal network, fertilization increased fungal community aggregation compared to the control treatment (N0), indicating that fertilization benefits network complexity and stability.

ZiPi analysis of key core species identified 675 bacterial core species and 117 fungal core species (**Supplementary Table S4**). The collinear network of bacteria included key core species such as *Pedomicrobium*, *Sphingomonas*, *Lysobacter*, *Methylophilaceae* (Proteobacteria), *Quadrisphaera* (Actinobacteria), and *Pedobacter* (Bacteroidota). The collinear network of fungi identified key ASVs, including core species such as *Diutina*, *Didymella*, *Pseudogymnoascus*, *Schizothecium*, *Cercophora* (Ascomycota), and *Basidioascus* (Basidiomycota).

### 3.5 Regulatory mechanisms of microbial diversity and yield

**Figure 5:**
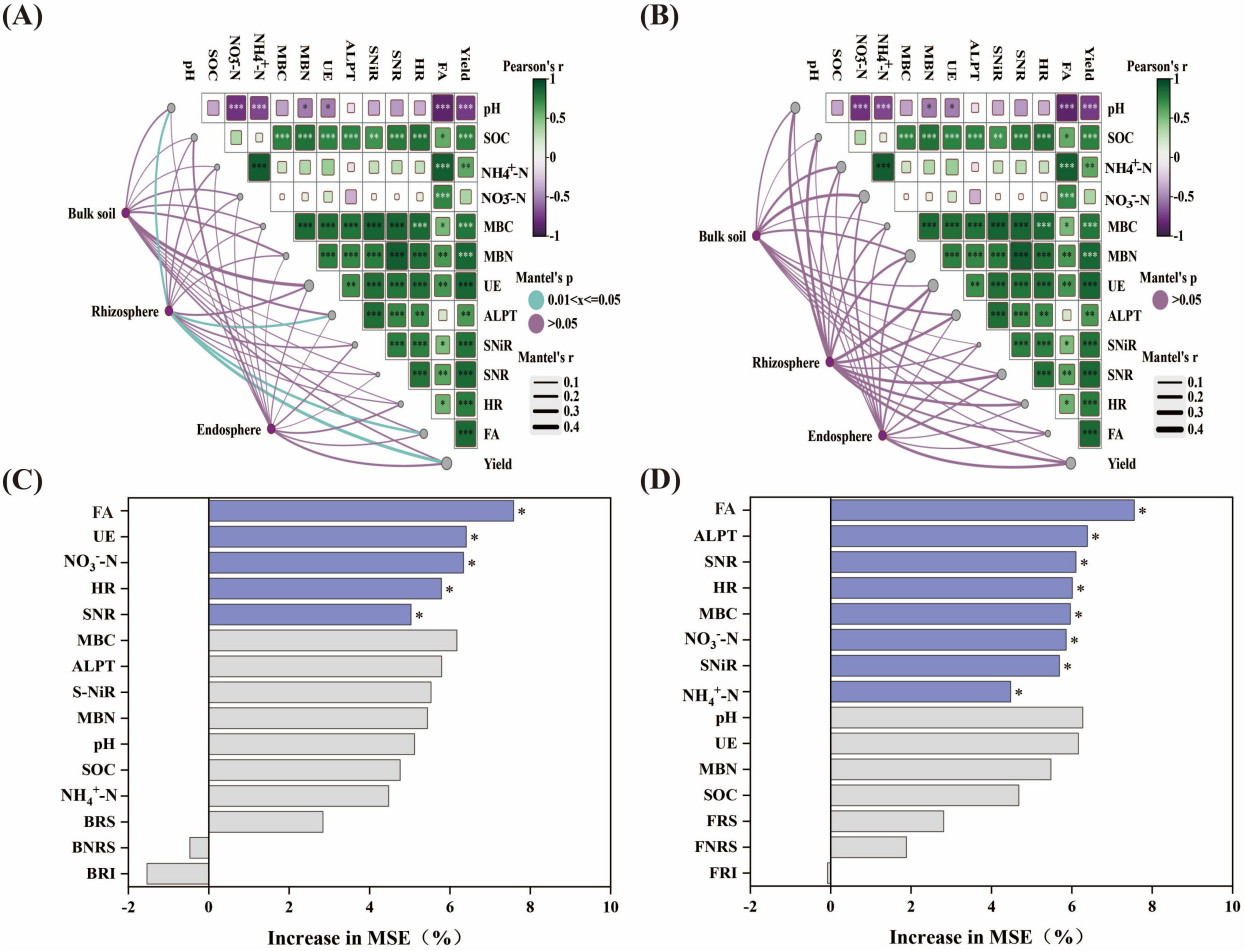
Mantel tests analyzed soil properties and the relationship between bacterial **(A)** and fungal **(B)** diversity and yield. The width of the line represents the partial Mantel statistic, and the color of the line represents the statistical significance based on 999 permutations. Pairwise correlations of environmental factors were represented by the Pearson correlation coefficient of color gradient. Random forests determine the effects of soil properties, bacterial **(C)**, and fungal **(D)** diversity on yield. * indicates *p* < 0.05. FA, Fertilizing amount; BNRS, Bulk soil bacteria; BRS, Rhizosphere bacteria; BRI, Endosphere bacteria; FNRS, Bulk soil fungi; FRS, Rhizosphere fungi; FRI, Endosphere fungi. ** means P<0.01; *** means P<0.001.

**Figure 6:**
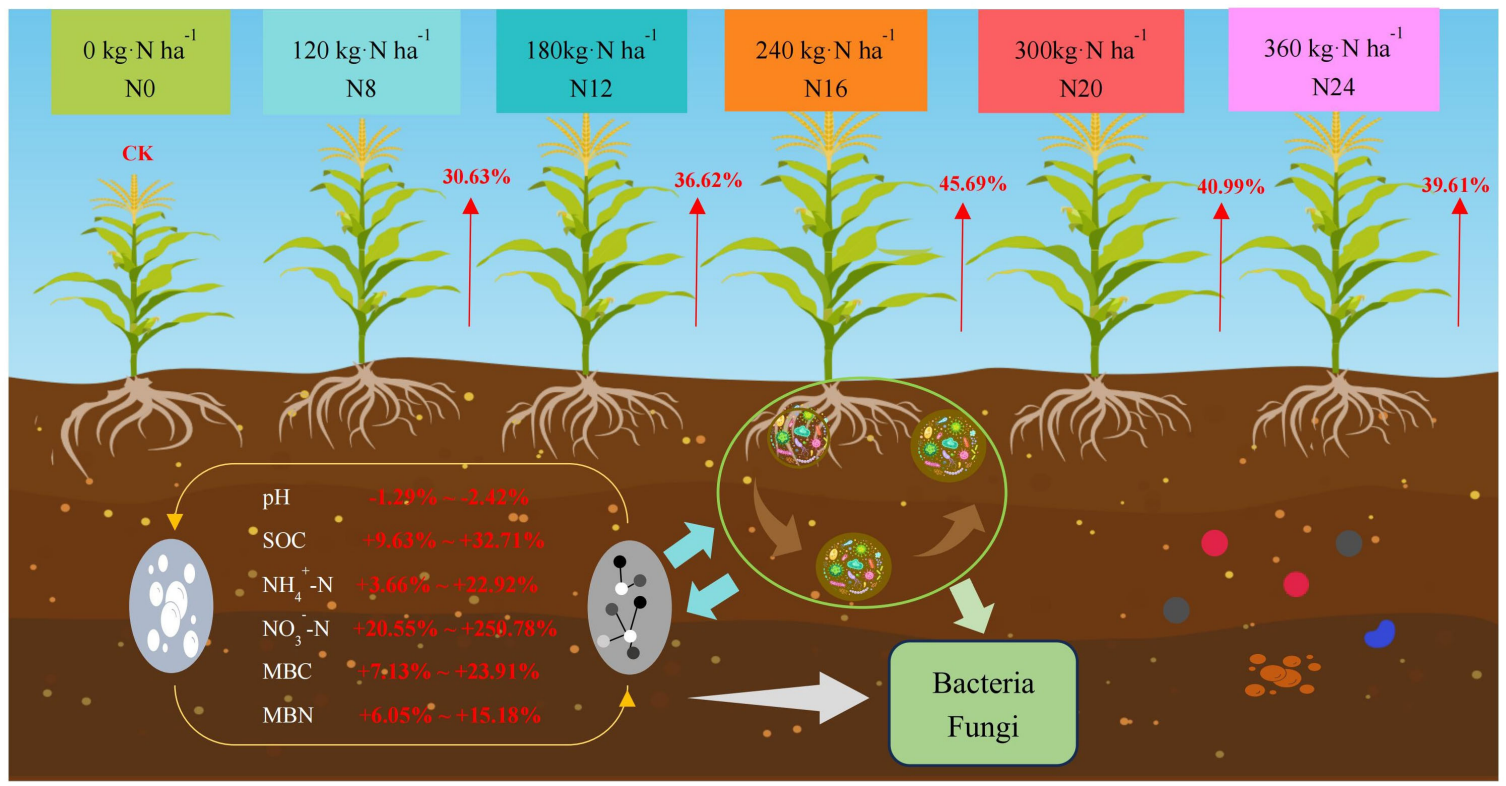
A model of soil physical and chemical properties, microbial community, and yield changes after adding nitrogen fertilizer.

The Mantel test results indicated that soil chemical properties, enzyme activity, and fertilizer amount were significantly correlated with yield (**Figure 5**). Bacterial microbial diversity across different ecological niches was correlated with FA, pH, UE, and yield. Notably, rhizosphere bacterial diversity was significantly correlated with yield and was also significantly influenced by pH, FA, and ALPT. Fungal microbial diversity across different ecological niches was correlated with pH, NO_3_^–^N, NH_4_^+^-N, MBN, SNR, UE, and yield, particularly the diversity of rhizosphere fungi. Random Forest analysis indicates that the primary factors influencing yield are FA, UE, NO_3_^–^N, HR, and SNR, It indicates that nitrogen application significantly affects the chemical properties and enzyme activities of the soil and further promotes the yield. These results provide a foundation for understanding how the application of slow-release nitrogen fertilizers improves soil physicochemical characteristics and alters soil microbial diversity and richness, thereby enhancing maize yield (**Figure 6**).

## 4 Discussion

### 4.1 The impact of fertilization on soil properties and crop yield

Nitrogen application can markedly influence soil physicochemical properties and enhance the soil microenvironment. Nitrogen application can lower soil pH, initially increase and then decrease the organic carbon mass fraction (Li et al., 2018), and simultaneously elevate nitrate nitrogen content in soil (Bian et al., 2022). In this study, nitrogen application led to a decrease in soil pH, showing a gradual declining trend with increased nitrogen application, whereas organic carbon exhibited an initial increase followed by a decrease. Soil enzyme activity is a crucial indicator of soil biochemical reaction activity and nutrient cycling (Wang and Allison, 2019). Urease, a key enzyme for urea hydrolysis, reflects soil nitrogen supply levels, with its activity significantly influenced by nitrogen fertilizer amount (Hu et al., 2023); increased nitrogen application markedly impacts soil urease activity. Soil nitrate reductase and nitrite reductase are crucial enzymes in soil denitrification, their activities indicate the extent of soil denitrification (Zheng et al., 2020). Hydroxylamine reductase facilitates the conversion of hydroxylamine to nitrite in soil (Wu et al., 2022). Previous studies have demonstrated that fertilization can significantly enhance soil enzyme activity and soil microbial biomass carbon and nitrogen content (Sun et al., 2023), aligning with our experimental results. Nonetheless, excessive nitrogen input can increase nutrient loss and inhibit microbial respiration, hindering the accumulation of soil microbial biomass (Wu et al., 2020). Thus, excess nitrogen inhibits microbial biomass carbon and nitrogen accumulation. Prior research (Chen P. et al., 2023) indicated that maize yield peaked at a nitrogen application rate of 278.4 kg·ha^-1^. In our study, yields were highest with moderate nitrogen application (N16), peaking at 240 kg·ha^-1^. This discrepancy may be closely tied to the spatial-temporal distribution of water, heat, and soil type. Additionally, we observed a significant correlation between microorganisms and maize yield, particularly rhizosphere microbial diversity changes, suggesting that microorganisms are potential factors influencing maize yield.

### 4.2 Effects of nitrogen application on microbial diversity

Soil microorganisms, including bacteria and fungi, play a pivotal role in facilitating soil organic matter turnover and enhancing soil nutrient mineralization rates (Qiu et al., 2022). Nitrogen application can augment soil bacterial diversity; particularly, optimal nitrogen application enhances bacterial diversity (Luo et al., 2022; Bandara et al., 2022), reduces fungal diversity (Wang, 2020), and boosts the diversity of the rhizosphere soil bacterial community (Zhao et al., 2019). In this study, nitrogen application elevated bacterial diversity in bulk soil, rhizosphere and endosphere, with microbial diversity progressively diminishing from bulk soil to endosphere. As nitrogen application rates increased, bacterial diversity initially rose and then declined. Excessive nitrogen fertilizer application rate can lower the soil C/N ratio, resulting in decreased soil microorganism number and activity due to carbon source scarcity, and a gradual decline in biological nitrogen fixation rates (Wu et al., 2024). Thus, over-fertilization will reduce soil microbial diversity. Various fertilizer types, application rates, geographical locations, and spatial positions significantly influence the microbial diversity of soil bacteria and fungi. This study revealed that with increased nitrogen application, the variations in fungi and bacteria across different spatial locations were more pronounced than those under varying nitrogen application rates (**Table 1**). This indicates that the soil environment is crucial in shaping the rhizosphere and internal microbial communities of plant hosts (Liu B. et al., 2023). Due to the rhizosphere effect, nitrogen application significantly impacted the microbial diversity of rhizosphere bacteria and fungi, enhancing bacterial diversity and reducing fungal diversity, whereas excessive nitrogen application inhibited microbial growth and affected microbial diversity.

### 4.3 The microbial community structure and co-occurrence network are influenced by fertilization

Microbial co-occurrence networks elucidate the intricate interactions among microorganisms, and the complexity of soil microbial networks actively contributes to maintaining ecosystem functions. Under conventional nitrogen application, the soil prokaryotic microbiome primarily exhibited higher richness, diversity, and a more complex network structure (Li et al., 2023). Fertilization typically alters microbial interactions, enhancing the complexity and connectivity of bacterial networks while reducing the complexity of fungal networks (Bai et al., 2022a), This is in line with the outcomes of this experiment. Compared with no nitrogen application, the application of slow-release nitrogen can enhance the complexity of bacterial and fungal networks and reduce the complexity of fungal communities (Barberán et al., 2012). This might be because the multi-year application of nitrogen offers a favorable environment for bacterial growth and promotes the interrelationships among species. Appropriate application of nitrogen fertilizer can increase network complexity, which is consistent with previous studies (Li et al., 2023), and the impact of fertilization on the rhizosphere microbiome is greater than that on bulk soil (Tian et al., 2021). This study also confirmed this conclusion. Due to the influence of the rhizosphere effect, the complexity of the bacterial network in the rhizosphere was higher than that in the bulk soil, while fungi demonstrated stronger complexity in both endosphere and bulk soil, indicating that bacteria and fungi were more sensitive to the rhizosphere effect.

Under varying nitrogen application treatments, the dominant bacterial phyla were Proteobacteria, Bacteroidota, Gemmatimonadota, and Actinobacteria. Concurrently, *Pedomicrobium*, *Sphingomonas*, *Lysobacter*, *Methylophilaceae* (Proteobacteria), *Quadrisphaera* (Actinobacteria), and *Pedobacter* (Bacteroidota) emerged as core species within the bacterial network. Proteobacteria primarily function in nutrient absorption and play a pivotal role in denitrification (Sun et al., 2020). Bacteroidota is essential in the nitrogen cycle within soil (Zhang et al., 2017), while Gemmatimonadota are closely associated with nitrogen fixation (Wei et al., 2020). Nitrogen application increases the abundance of these dominant phyla and promotes nutrient cycling (Zhang et al., 2018). The dominant fungal phyla were Ascomycota and Basidiomycota, with *Diutina*, *Didymella*, *Pseudogymnoascus*, *Schizothecium*, *Cercophora* (Ascomycota), and *Basidioascus* (Basidiomycota) also identified as core species within the fungal network. Ascomycota and Basidiomycota are vital decomposers of complex compounds, crucially involved in decomposing plant residues and degrading straw residues (Liu et al., 2021). Nitrogen application increases the abundance of Ascomycota and Basidiomycota in fungi (Zhang et al., 2024).

These dominant phyla facilitate root nutrient absorption, enhance nitrogen uptake and utilization, reduce volatilization, improve nitrogen transport efficiency, and provide sufficient substrates for organic synthesis. Thus, the dominant bacterial and fungal species in this study were instrumental in reshaping the soil microenvironment and enhancing crop yield. The soil enzymes they secrete also influence the soil’s physical and chemical properties, positively responding to appropriate nitrogen fertilizer application rate. This suggests that appropriate nitrogen application can alter soil microbial community composition and diversity, thereby enhancing soil microbial function and boosting crop yield (Shu et al., 2022). Although fertilization decreases the complexity of bacterial networks, it increases the abundance of beneficial bacteria and promotes nutrient absorption, thereby offering a theoretical basis for enhancing maize yield.

## 5 Conclusion

The interplay between nitrogen application and rhizosphere selection markedly influenced multiple microbial attributes, including α-diversity, community architecture, and microbial network configuration. Findings from this study revealed that nitrogen application substantially altered soil physicochemical properties, augmented microbial biomass carbon and nitrogen, enhanced soil enzyme activity, and increased microbial diversity. Moreover, it had a more pronounced impact on rhizosphere microorganisms compared to bulk soil and endosphere microorganisms. Dominant microbial taxa such as *Sphingomonas*, *Lysobacter*, and *Methylophilaceae* facilitate organic matter decomposition, enhance nitrogen transport efficiency, and synergize crop growth. Slow-release nitrogen fertilizers increased the network complexity of bacterial communities while reducing the stability and complexity of fungal networks, indicating that bacterial communities exhibited superior coordination of the soil microenvironment, thereby further boosting yield. Notably, the microbial diversity within the bacterial rhizosphere exerted a more substantial influence on yield compared to fungal diversity. Additionally, the quantity of fertilizer application, along with nitrate nitrogen, nitrate reductase, and hydroxylamine reductase, were pivotal determinants of yield. In particular, soil microbiological traits and soil chemical traits were higher at a moderate nitrogen fertilizer application rate (180 – 240 kg·ha^-1^), which increased inter-root microbial diversity, facilitated soil nutrient cycling, and drove increased yields. These findings have significantly broadened our comprehension of soil microenvironment regulation from a microbial perspective. In summary, the deployment of slow-release nitrogen fertilizers can markedly modulate the microbial community architecture across various ecological niches in maize fields, particularly enhancing the microbial diversity and network structure within the rhizosphere.

## Data Availability

The obtained sequences were submitted to the NCBI Sequence Read Archive (SRA) with BioProject number PRJNA1142092.
