# Supplementary material for: Slow-release nitrogen fertilizer application regulated rhizosphere microbial diversity to increase maize yield
Source: Front Plant Sci. 2024 Nov 22;15:1481465. doi: 10.3389/fpls.2024.1481465 (PMC11620899; doi:10.3389/fpls.2024.1481465)
Supplement: Supplementary file 1 [file DataSheet1.docx]

Supplementary Material

Slow-release nitrogen fertilizer application regulated rhizosphere microbial diversity to increase maize yield

Tiantian Meng^1^, Jingjing Shi^4^, Xiangqian Zhang^2,3*^, Xiaoqing Zhao^2,3^, Dejian Zhang^4^, Liyu Chen^2,3^, Zhanyuan Lu^1,2,3*^, Yuchen Cheng^2,3^, Yonghe Hao^5^, Yu Wang^5*^

*** Correspondence:**

Xiangqian Zhang
[zhangxiangqian_2008@126.com](mailto:zhangxiangqian_2008@126.com)

Zhanyuan Lu
[lzhy2811@163.com](mailto:lzhy2811@163.commail@uni.edu)

# Supplementary Data

Supplementary Material should be uploaded separately on submission. Please include any supplementary data, figures and/or tables.

Supplementary material is not typeset so please ensure that all information is clearly presented, the appropriate caption is included in the file and not in the manuscript, and that the style conforms to the rest of the article.

# Supplementary Figures and Tables

For more information on Supplementary Material and for details on the different file types accepted, please see [here](https://www.frontiersin.org/guidelines/author-guidelines#supplementary-material).

## Supplementary Figures
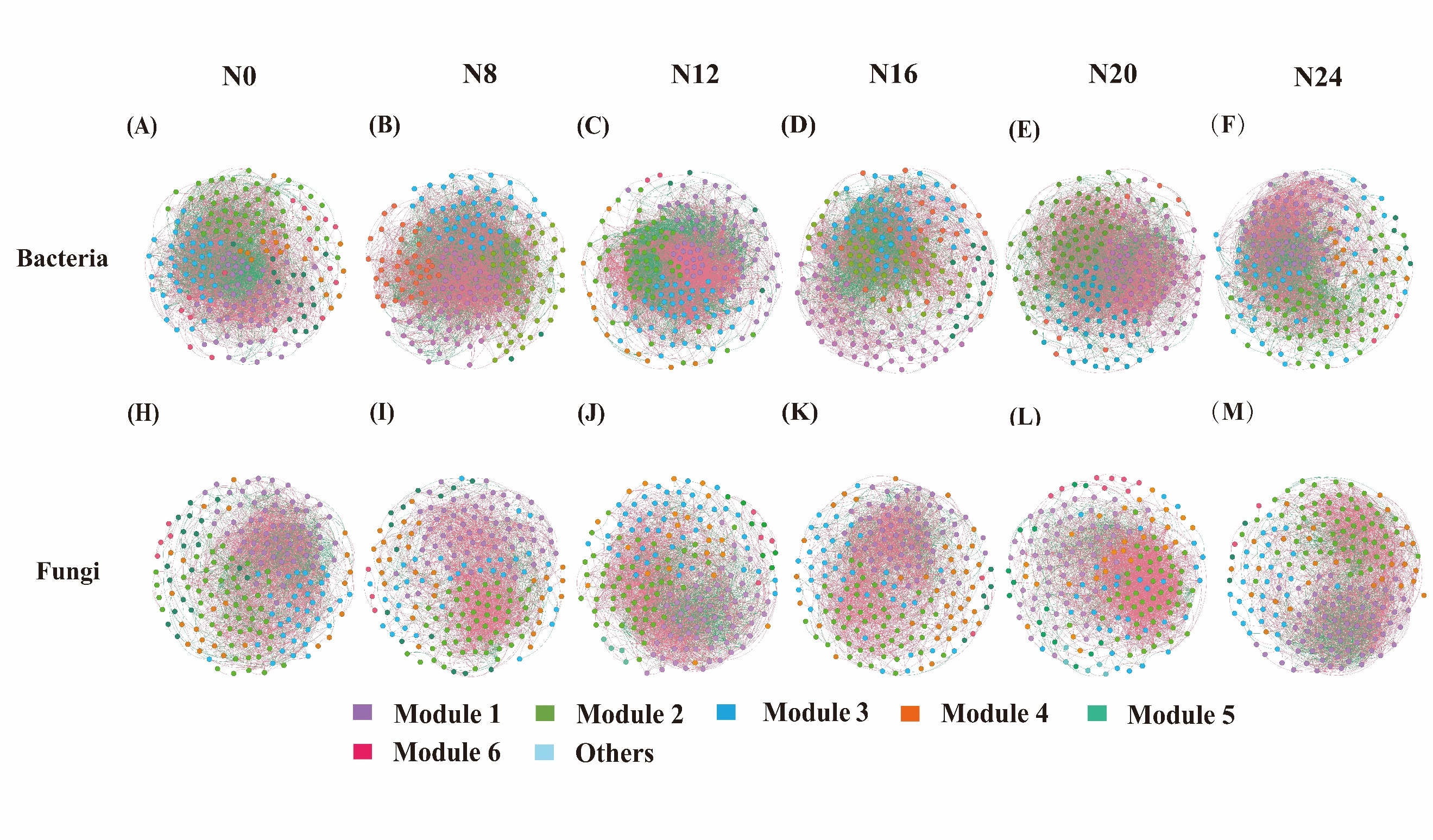


**Supplementary Figure 1.** The ASV collinearity of bacteria（A-F） and fungal（H-M） communities was assessed under different nitrogen application rate


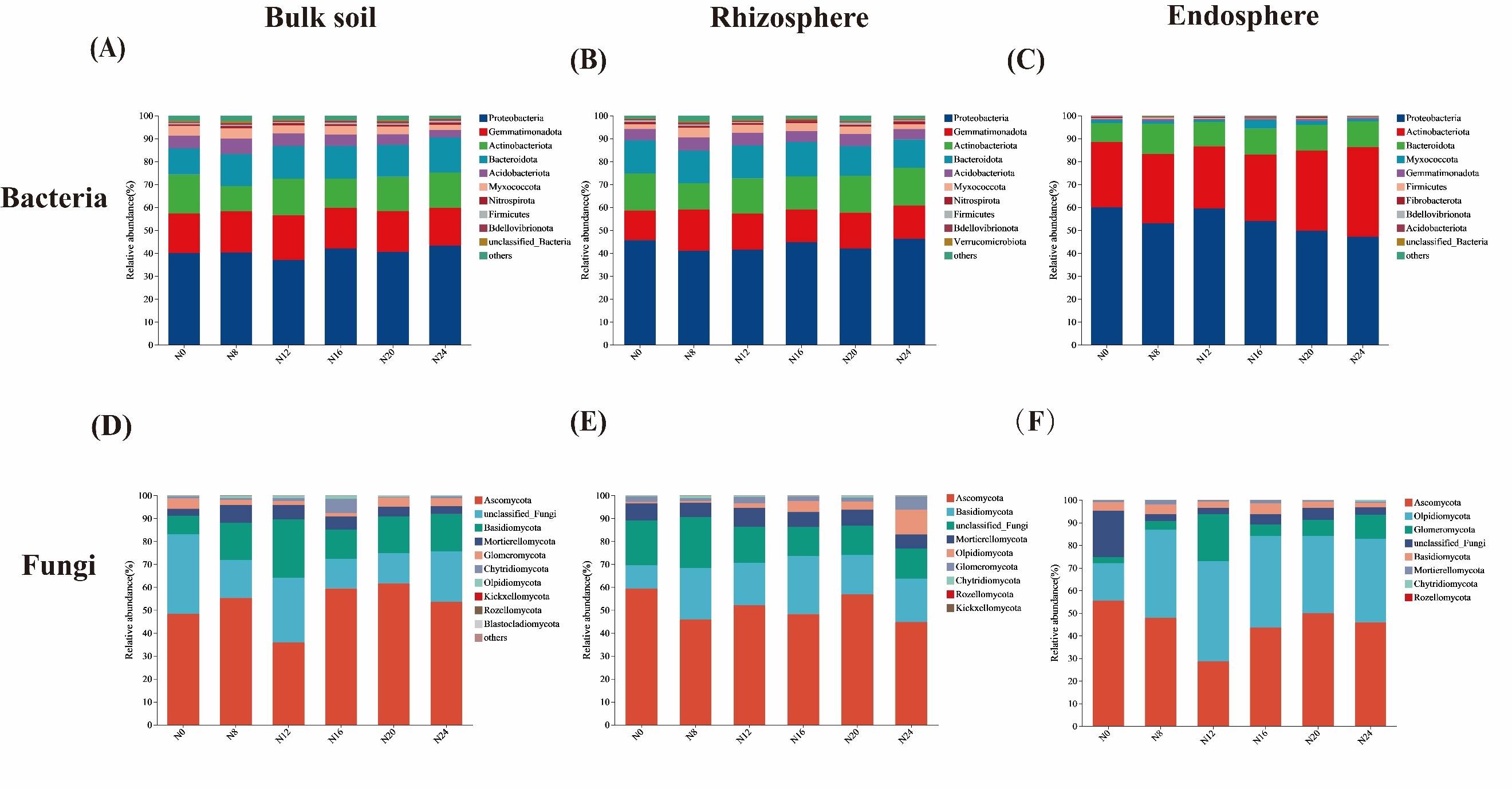


**Supplementary Figure 2.** The relative abundance of major taxonomic groups at the phylum level for bacteria (A-C) and fungi (D-F). The width of the bars from each phylum indicated the relative abundance of the phylum.

## Supplementary Table

**Supplementary Table 1**. Response of α-diversity to increasing nitrogen fertilizer application rate

| **Type of microorganism** | **Nitrogen fertilizer application rate** | **Bulk soil** | | **Rhizosphere** | | **Endosphere** | |
| --- | --- | --- | --- | --- | --- | --- | --- |
|  |  | **Chao index** | **Shannon index** | **Chao index** | **Shannon index** | **Chao index** | **Shannon index** |
| Bacteria | N0 | 1138.47±207.26a | 6.5±0.15a | 720.75±23.84c | 6.05±0.03c | 387.44±121.04a | 4.95±0.24a |
|  | N8 | 1228.23±101.95a | 6.59±0.07a | 1469.28±219.73a | 6.75±0.15a | 652.9±123.92a | 5.31±0.23a |
|  | N12 | 1267.07±293.07a | 6.57±0.22a | 1452.37±270.45a | 6.7±0.16a | 538.91±151.74a | 5.04±0.27a |
|  | N16 | 1086.5±324.04a | 6.43±0.33a | 846.71±61.76b | 6.24±0.08b | 649.06±103.66a | 5.37±0.01a |
|  | N20 | 1317.34±237.58a | 6.61±0.15a | 1258.16±210.03ab | 6.59±0.14ab | 604.25±64.81a | 5.21±0.25a |
|  | N24 | 925.23±95.08a | 6.35±0.11a | 892.52±229.97b | 6.21±0.27c | 565.27±32.03a | 5.18±0.27a |
| Fungi | N0 | 396.56±12.41a | 4.44±0.22a | 379.9±3.75b | 4.4±0.08a | 179±15.87bc | 2.79±0.15a |
|  | N8 | 389.81±1.58a | 4.5±0.15a | 469.24±7.27b | 4.02±0.67a | 191.41±4.15a | 2.64±0.27a |
|  | N12 | 411.52±28.8a | 4.09±0.46a | 382.9±4.81a | 3.93±0.24a | 170.15±2.01bc | 2.62±0.34a |
|  | N16 | 364.55±13.12a | 4.15±0.14a | 407.82±14.63bc | 4.51±0.06a | 184.11±26.17b | 2.92±0.37a |
|  | N20 | 358.71±17.5a | 4.09±0.48a | 381.84±24.22c | 4.55±0.1a | 189.51±27.79bc | 2.93±0.16a |
|  | N24 | 407.4±7.11a | 4.28±0.08a | 369.42±15.85ab | 4.29±0.38a | 205.66±36.69c | 2.95±0.43a |

**Supplementary Table 2** Average network distance, modularity index, clustering coefficient and graph density of network analysis graphs of bacteria and fungi at different locations

| **Microbial species** | **Ecological niche** | **Nodes** | **Edges** | **Positive（%）** | **Negative（%）** | **Average network distance** | **Modularity index** | **Clustering coefficient** | **Average degree** |
| --- | --- | --- | --- | --- | --- | --- | --- | --- | --- |
| Bacteria | Bulk soil | 200 | 1131 | 55.08 | 44.92 | 2.646 | 0.399 | 0.248 | 11.31 |
|  | Rhizosphere | 200 | 1082 | 62.57 | 37.43 | 2.739 | 0.362 | 0.234 | 10.82 |
|  | Endosphere | 200 | 1099 | 64.33 | 35.67 | 2.68 | 0.428 | 0.261 | 10.99 |
| Fungi | Bulk soil | 199 | 1534 | 60.82 | 39.18 | 2.558 | 0.349 | 0.344 | 15.47 |
|  | Rhizosphere | 199 | 1815 | 60.5 | 39.5 | 2.346 | 0.349 | 0.349 | 18.24 |
|  | Endosphere | 199 | 999 | 72.97 | 27.03 | 2.833 | 0.29 | 0.29 | 10.04 |

**Supplementary Table 3** Average network distance, modularity index, clustering coefficient and graph density of network analysis graphs of bacteria and fungi under different nitrogen application rate

| **Microbial species** | **Treatment** | **Nodes** | **Edges** | **Positive（%）** | **Negative（%）** | **Average network distance** | **Modularity index** | **Clustering coefficient** | **Average degree** |
| --- | --- | --- | --- | --- | --- | --- | --- | --- | --- |
|  | N0 | 196 | 4485 | 55.81 | 44.19 | 2.101 | 0.217 | 0.627 | 45.765 |
| **Bacteria** | N8 | 197 | 5263 | 52.38 | 47.62 | 1.957 | 0.183 | 0.656 | 53.431 |
|  | N12 | 198 | 5699 | 52.48 | 47.52 | 2.021 | 0.163 | 0.604 | 57.566 |
|  | N16 | 200 | 4281 | 57.86 | 42.14 | 2.146 | 0.214 | 0.595 | 42.81 |
|  | N20 | 198 | 5274 | 51.97 | 48.03 | 1.969 | 0.201 | 0.635 | 53.273 |
|  | N24 | 200 | 4207 | 55.17 | 44.83 | 2.161 | 0.234 | 0.587 | 42.07 |
| **Fungi** | N0 | 199 | 2941 | 72.87 | 27.13 | 2.37 | 0.279 | 0.563 | 29.558 |
|  | N8 | 199 | 2447 | 79.98 | 20.02 | 2.484 | 0.338 | 0.518 | 24.493 |
|  | N12 | 200 | 3079 | 74.44 | 25.56 | 2.305 | 0.289 | 0.548 | 30.79 |
|  | N16 | 198 | 2661 | 71.7 | 28.3 | 2.399 | 0.329 | 0.52 | 26.879 |
|  | N20 | 199 | 2954 | 77.35 | 22.65 | 2.48 | 0.258 | 0.565 | 29.688 |
|  | N24 | 198 | 3075 | 72.91 | 27.09 | 2.286 | 0.373 | 0.569 | 31.061 |

**Supplementary Table 4** Top 30 Key species of network analysis graphs of bacteria and fungi under different nitrogen application rate

| **Microbial type** | **ASV** | **Phylum** | **Family/Genus** | **Degree** | **Type** |
| --- | --- | --- | --- | --- | --- |
| **Bacteria** | ASV13842 | Proteobacteria | Pedomicrobium | 118 | Connectors |
|  | ASV3670 | Gemmatimonadota | norank_S0134_terrestrial_group | 117 | Connectors |
|  | ASV4997 | Actinobacteriota | unclassified_Intrasporangiaceae | 116 | Connectors |
|  | ASV656 | Actinobacteriota | Quadrisphaera | 115 | Connectors |
|  | ASV21754 | Actinobacteriota | norank_67-14 | 114 | Connectors |
|  | ASV6293 | Myxococcota | norank_Myxococcaceae | 113 | Connectors |
|  | ASV55 | Proteobacteria | Sphingomonas | 112 | Connectors |
|  | ASV17637 | Proteobacteria | Lysobacter | 111 | Connectors |
|  | ASV909 | Proteobacteria | Methylophilaceae | 110 | Connectors |
|  | ASV21258 | Bacteroidota | Pedobacter | 109 | Connectors |
|  | ASV940 | Bacteroidota | KD3-93 | 108 | Connectors |
|  | ASV16773 | Proteobacteria | Steroidobacter | 107 | Connectors |
|  | ASV16769 | Patescibacteria | norank_Saccharimonadales | 106 | Connectors |
|  | ASV11626 | Gemmatimonadota | Gemmatimonadaceae | 105 | Connectors |
|  | ASV6106 | Proteobacteria | Bosea | 104 | Connectors |
|  | ASV815 | Bacteroidota | AKYH767 | 103 | Connectors |
|  | ASV3149 | Proteobacteria | Steroidobacter | 102 | Connectors |
|  | ASV3087 | Bacteroidota | Chryseolinea | 101 | Connectors |
|  | ASV1177 | Proteobacteria | Rhizobiaceae | 100 | Connectors |
|  | ASV4544 | Bacteroidota | Parasegetibacter | 99 | Connectors |
| **Fungi** | ASV2169 | unclassified_Fungi | unclassified_Fungi | 51 | Connectors |
|  | ASV4236 | Ascomycota | Diutina | 50 | Connectors |
|  | ASV2246 | Ascomycota | Didymella | 49 | Connectors |
|  | ASV1199 | Basidiomycota | Basidioascus | 48 | Connectors |
|  | ASV2579 | Ascomycota | Pseudogymnoascus | 47 | Connectors |
|  | ASV2000 | unclassified_Fungi | unclassified_Fungi | 46 | Connectors |
|  | ASV1145 | Ascomycota | Cercophora | 45 | Connectors |
|  | ASV1253 | unclassified_Fungi | unclassified_Fungi | 44 | Connectors |
|  | ASV978 | unclassified_Fungi | unclassified_Fungi | 43 | Connectors |
|  | ASV5233 | Ascomycota | Striaticonidium | 42 | Connectors |
|  | ASV2468 | unclassified_Fungi | unclassified_Fungi | 41 | Connectors |
|  | ASV4604 | Kickxellomycota | Linderina | 40 | Connectors |
|  | ASV413 | unclassified_Fungi | unclassified_Fungi | 39 | Connectors |
|  | ASV1858 | Ascomycota | Schizothecium | 38 | Connectors |
|  | ASV822 | Basidiomycota | Coprinellus | 37 | Connectors |
|  | ASV2952 | Glomeromycota | Diversispora | 36 | Connectors |
|  | ASV733 | Ascomycota | Sphaeronaemella | 35 | Connectors |
|  | ASV1538 | unclassified_Fungi | unclassified_Fungi | 34 | Connectors |
|  | ASV387 | Ascomycota | unclassified_Nectriaceae | 33 | Connectors |
|  | ASV1812 | Ascomycota | Chrysosporium | 32 | Connectors |
